# Corn straw-saccharification fiber improved the reproductive performance of sows in the late gestation and lactation via lipid metabolism

**DOI:** 10.3389/fnut.2024.1370975

**Published:** 2024-03-28

**Authors:** Mengjie Liu, Chaoqi Liu, Jiajia Shi, Ping Wang, Juan Chang, Xiaoxiang Xu, Lijun Wang, Sanjun Jin, Xinxin Li, Qingqiang Yin, Qun Zhu, Xiaowei Dang, Fushan Lu

**Affiliations:** ^1^College of Animal Science and Technology, Henan Agricultural University, Zhengzhou, China; ^2^Shanghai Skin Disease Hospital, School of Medicine, Tongji University, Shanghai, China; ^3^Henan Delin Biological Product Co. Ltd., Xinxiang, China; ^4^Henan Puai Feed Co. Ltd., Zhoukou, China

**Keywords:** corn straw, saccharification, sow, reproductive performance, nutrient digestibility, metabolomics

## Abstract

With the development of animal husbandry, the shortage of animal feedstuffs has become serious. Dietary fiber plays a crucial role in regulating animal health and production performance. The aim of this study was to investigate the effects of three kinds of corn straw-saccharification fibers (CSSF) such as high-fiber and low-saccharification (HFLS), medium-fiber and medium-saccharification (MFMS), low-fiber and high-saccharification (LFHS) CSSF on the reproductive performance of sows. Thirty-two primiparous Yorkshire sows were randomly assigned to 4 groups, 8 sows for each group. Group A was the basal diet as the control group; groups B – D were added with 6% HFLSCSSF, 6% MFMSCSSF and 6% LFHSCSSF to replace some parts of corn meal and wheat bran in the basal diet, respectively. The experimental period was from day 85 of gestation to the end of lactation (day 25 post-farrowing). The results showed that 6% LFHSCSSF addition significantly increased number of total born (alive) piglets, litter weight at birth (*p* < 0.05), whereas three kinds of CSSF significantly decreased backfat thickness of sows during gestation (*p* < 0.001), compared with the control group. Furthermore, CSSF improved the digestibility of crude protein, ether extract and fiber for sows. In addition, the levels of total cholesterol, total triglycerides, and high-density lipoprotein cholesterol in serum of sows were decreased by different kinds of CSSF. Further analysis revealed that CSSF regulated lipid metabolism through adjusting the serum metabolites such as 4-pyridoxic acid, phosphatidyl cholines and L-tyrosine. In summary, CSSF addition to the diets of sows during late gestation and lactation regulated lipid metabolism and improved reproductive performance of sows. This study provided a theoretical basis for the application of corn straw in sow diets.

## 1 Introduction

With the increasing demand for food, agriculture and animal husbandry are expanding rapidly (1). It is estimated that the livestock and poultry production will be increased by 21% until 2025 (2), indicating a significant increase in the demand for feedstuffs. As a result, the food competition between humans and animals is becoming intense (1). Therefore, it is crucial to develop new feed resources to replace the traditional feedstuffs. Lignocellulose with approximately 75% polysaccharide is a low-cost renewable resource composed of cellulose, hemicellulose, and lignin, which has the potential to be converted to animal feed (3–5).

Corn straw is one of the agricultural by-products, with a global annual production of approximately 1.639 billion tons in 2022 (6). It is rich in lignocellulose, but the complex structure of lignocellulose inhibit enzymatic saccharification, resulting in low utilization rates and causing serious waste and environmental pollution (7). Many reports discovered that corn straw could be hydrolyzed to monosaccharides by physical, chemical, microbial and enzymatic methods, and subsequently developed into animal feed materials (8), which were widely used in ruminants (9–11). However, due to differences in the digestive systems of monogastric and ruminant animals (12), the utilization of high-fiber feed in monogastric animals is irregular (13). Previous studies demonstrated that the lignocellulose in corn straw was degraded by physical, chemical, and microbial treatments to be used as a substitute for energy feedstuffs in the diets of monogastric animals such as chickens and pigs without reducing their production performances (14–16).

More and more studies have proved that dietary fiber reduces obesity and improves health by regulating lipid metabolism (17–19). Currently, the regulation of sow health by dietary fiber has received widespread attention. The previous research indicated that dietary fiber increased the satiety of sows (20) and improved their reproductive performance (21). Furthermore, the addition of wheat bran to the diet of sows was found to improve pregnancy weight gain and lipid metabolism (22). However, due to the different physicochemical properties of different fibers, the responses to high-fiber diets may be different (23). In our previous study, we prepared different kinds of corn straw-saccharification fiber (CSSF) through a combination of physical, chemical and enzymatic methods (4), converting the lignocellulose of corn straw to low-molecular-weight carbohydrates as a source of energy for monogastric animals, aiming to solve feed shortages and regulate lipid metabolism. So far, there is limited research on the use of CSSF as a feed and dietary fiber source for sows in late gestation and lactation. The purpose of this study was to investigate the effects of different kinds of CSSF on the reproductive performance of sows during the late gestation and lactation as well as the associated changes in lipid metabolism, in order to provide a theoretical basis for the development and application of straw resources in sow production.

## 2 Materials and methods

All protocols in this experiment were approved by the Animal Care and Use Ethics Committee of Henan Agricultural University (SKLAB-B-2010-003-01), and conducted in compliance with the relevant regulations and guidelines.

### 2.1 Preparation and nutritional composition of experimental diets

#### 2.1.1 CSSF preparation

Corn straw was collected from a farm (Henan, China). Cellulase (322.37 filter-paper unit (FPU)/g) and β-glucanase (10,000 U/g) were purchased from Ningxia Xiasheng Industrial Group Co., Ltd. (Ningxia, China). NaOH, CaO, HCl and H_2_SO_4_ were purchased from Damao Chemical Reagent Factory (Tianjin, China). The air-dried corn straw was crushed to 1 mm by a hammer mill and stored at room temperature. The corn straw was mixed with alkaline solution (0.8% NaOH +1.2% CaO, w/v) at a ratio of 5:1 (w/v), steamed for 1.0 h, and the pH was adjusted to 4.8 by HCl and H_2_SO_4_, then dried and pulverized to obtain high-fiber and low-sacchrification CSSF (HFLSCSSF). On the basis of HFLSCSSF, cellulase (15 FPU/g biomass) and β-glucanase (2,000 U/g biomass) were added, and the mixture was evenly stirred; the straw was enzymolized at 50°C for 12 h, then dried and ground to obtain medium-fiber and medium-sacchrification CSSF (MFMSCSSF). On the basis of HFLSCSSF, cellulase (28.12 FPU/g biomass) and β-glucanase (6,600 U/g biomass) were added, and the mixture was evenly stirred; the straw was enzymolized at 50°C for 40 h, then dried and ground to obtain low-fiber and high-sacchrification CSSF (LFHSCSSF). The basal nutrient composition of different kinds of CSSF was shown in Table 1.

**Table 1:** Nutrient compositions of different kinds of CSSF (%, dry matter basis).

Item	Raw corn straw	HFLSCSSF^1^	MFMSCSSF^2^	LFHSCSSF^3^
Cellulose	31.59	24.61	9.54	5.56
Hemicellulose	28.84	11.29	8.01	4.61
Lignin	4.06	2.18	1.42	1.37
Reducing sugar, mg/g	23.38	37.57	280.99	388.93
Crude protein	6.71	4.72	6.69	9.54
Ether extract	1.05	1.14	2.02	3.42
Digestible energy, MJ/kg	1.42	6.81	7.54	7.00

^1^HFLSCSSF: high-fiber and low-saccharification CSSF. ^2^MFMSCSSF: medium-fiber and medium-saccharification CSSF. ^3^LFHSCSSF: low-fiber and high-saccharification CSSF.

#### 2.1.2 Determination of digestible energy (DE) for CSSF

The digestible energy of CSSF was determined with a simulated digestive system (SDS-II, Hunan Zhongben Intelligent Technology Development Co., Ltd., Changsha, China) according to the previous study *in vitro* (24). The simulated gastric juice consisted of pepsin (890 U/mL), pH was adjusted to 2.0 with 2 mol/L hydrochloric acid (HCl). The gastric buffer solution was prepared with 80.6 mmol/L NaCl and 6 mmol/L KCl, and pH was adjusted to 2.0 with 2 mol/L HCl. The small intestinal buffer was composed of 30 mmol/L Na_2_HPO_4_, 170 mmol/L NaH_2_PO_4_, 98.8 mmol/L NaCl, 16.0 mmol/L KCl, 0.24 g/L penicillin sodium (1,600,000 units), and pH was adjusted to 6.44 using 1 mol/L NaOH. The concentrated simulated small intestinal fluid was composed of 1,323 U/mL trypsin, 166 U/mL chymotrypsin and 4,239 U/mL amylase. The large intestinal buffer was composed of 30 mmol/L Na_2_HPO_4_, 170 mmol/L NaH_2_PO_4_, 102.5 mmol/L NaCl, 12.2 mmol/L KCl, 0.24 g/L penicillin sodium (1,600,000 units), and pH was adjusted to 6.42 using 1 mol/L NaOH. The concentrated simulated large intestinal fluid was prepared with 491 U/mL trypsin, 62 U/mL chymotrypsin, 1,572 U/mL amylase and 0.77 U/mL cellulase. The procedures were as follows: 2 g CSSF samples and 20 mL simulated gastric juice were added into a dialysis bag, which was put in a digestive chamber. After the gastric buffer solution circulated the dialysis bag for 3 h in the chamber, the buffer solution was emptied. Then, the small intestinal buffer was pumped into the digestive chamber to circulate the dialysis bag. 1 h later, 2 mL concentrated simulated small intestinal fluid was injected into the dialysis bag through a peristaltic pump to simulate small intestinal digestion. After the small intestinal digestion was conducted for 5 h, the buffer in the digestion chamber was emptied, and the large intestinal buffer was pumped into the chamber to circulate the dialysis bag. 0.1 h later, 2 mL concentrated simulated large intestinal fluid was pumped into the dialysis bag to react for 20.9 h. After digestion was finished, the buffer was emptied and the residue in the dialysis bag was washed 6 times with deionized water to remove the by-products, then the undigested residue was degreased and dried. The gross energy (GE) of the CSSF sample and the degreased residue were determined by an automatic oxygen bomb calorimeter (IKA Instrument Co., Staufen, Germany). The digestible energy = (CSSF weight × GE of CSSF − degreased residue weight × GE of degreased residue)/CSSF weight.

#### 2.1.3 Animals and experimental diets

Thirty-two primiparous Yorkshire sows with good health and similar body condition were randomly assigned to 4 groups, 8 sows in each group. Group A was the basal diet as the control group; groups B – D were added with 6% HFLSCSSF, MFMSCSSF and LFHSCSSF to replace corn meal and wheat bran in the basal diet, respectively. The experimental diets were formulated according to pig nutrient requirements of NRC (25) (Table 2).

**Table 2:** Diet compositions and nutrient levels of sows (%, dry matter basis).

Item	Gestation				Lactation			
	A	B	C	D	A	B	C	D
Corn meal	54.39	52.99	53.35	53.46	58.34	54.587	55.407	56.137
Wheat bran	25.18	20.19	20.12	20.58	9.04	7.43	6.83	6.45
Soybean meal	11.81	13.14	12.83	12.24	5.88	6.27	6.04	5.63
Extruded soybean	5.07	5.07	5.07	5.07	12.20	12.20	12.20	12.20
HFLSCSSF^1^		6.00				6.00		
MFMSCSSF^2^			6.00				6.00	
LFHSCSSF^3^				6.00				6.00
Monocalcium phosphate	0.60	0.88	0.85	0.85				0.05
CaCO_3_	1.38	0.53	0.57	0.57	0.68			
NaCl	0.35				0.35			
Lysine	0.21	0.19	0.20	0.22			0.01	0.02
Methionine	0.01	0.01	0.01	0.01	0.01	0.013	0.013	0.013
Premix^4^	1.00	1.00	1.00	1.00				
Concentrate^5^					13.50	13.50	13.50	13.50
Total	100	100	100	100	100	100	100	100
Nutrient levels^6^								
CP	17.94	17.11	17.25	18.07	18.66	19.1	18.89	18.47
DE (MJ/kg)	14.04	13.89	13.95	13.94	14.84	14.52	14.48	14.51
Ca	0.76	0.76	0.88	0.8	1.05	1.19	1.19	1.04
P	0.63	0.6	0.6	0.62	0.49	0.48	0.45	0.46
Lysine	0.81	0.8	0.81	0.8	1.3	1.31	1.3	1.3
Methionine	0.24	0.24	0.24	0.24	0.23	0.23	0.23	0.23
NDF	30.29	30.98	40.43	41.01	39.13	37.56	39.49	34.03
ADF	6.04	6.74	5.57	5.15	4.89	7.97	5.45	4.77
Cellulose	5.38	6.05	4.95	4.6	4.29	6.49	4.51	4.14
Hemicellulose	24.27	24.22	34.86	35.85	34.22	29.57	34.04	29.26

CP, crude protein; DE, digestible energy; Ca, calcium; P, phosphorus; NDF, neutral detergent fiber; ADF, acid detergent fiber. ^1^HFLSCSSF: high-fiber and low-saccharification CSSF. ^2^MFMSCSSF: medium-fiber and medium-saccharification CSSF. ^3^LFHSCSSF: low-fiber and high-saccharification CSSF. ^4^Premix in diet per kilogram: VA 7000 IU; VD3 2,600 IU; VE 800 IU; VK3 1.30 mg; VB1 3.0 mg; VB2 15.0 mg; Nicotinic acid 35.0 mg; Choline 1.50 g; Pantothenate: 30.0 mg; VB12 0.03 mg; VB6 2.50 mg; Biotin 0.60 mg; Folic acid 3.30 mg; Cu (as copper sulfate) 16.0 mg; Fe (as ferrous sulfate) 160.0 mg; Mn (as manganese sulfate) 50.0 mg; Zn (as zine oxide) 160.0 mg; I (as calcium iodate) 0.30 mg; Se (as sodium selenite) 0.30 mg. ^5^Concentrate per kilogram: CP 38.75 g; Ca 5.96 g; P 2.58 g; Lysine 6.48 g; VA 7000 IU; VD3 2,600 IU; VE 800 IU; VK3 1.30 mg; VB1 3.0 mg; VB2 15.0 mg; Nicotinic acid 35.0 mg; Choline 1.50 g; Pantothenate: 30.0 mg; VB12 0.03 mg; VB6 2.50 mg; Biotin 0.60 mg; Folic acid 3.30 mg; Cu (as copper sulfate) 16.0 mg; Fe (as ferrous sulfate) 160.0 mg; Mn (as manganese sulfate) 50.0 mg; Zn (as zine oxide) 160.0 mg; I (as calcium iodate) 0.30 mg; Se (as sodium selenite) 0.30 mg. ^6^The CP, DE, Ca, P, NDF, ADF, cellulose and hemicellulose levels were measured, whereas the others were calculated. A: the basal diet. B: 6% HFLSCSSF added in the basal diet to replace corn meal and wheat bran. C: 6% MFMSCSSF added in the basal diet to replace corn meal and wheat bran. D: 6% LFHSCSSF added in the basal diet to replace corn meal and wheat bran.

### 2.2 Experimental design

From day 85 to 106 of gestation, each sow was housed in an individual gestation stall (2.1 m × 0.6 m), and then transferred to an individual farrowing bed (2.2 m × 3.6 m) on day 107 of gestation. During late gestation, the sows in each group were fed with 3.0 kg/d gestation diet from day 85 to 100, 4.5 kg/d gestation diet from day 101 to 113, and 2.0 kg/d gestation diet from day 114 to parturition. During lactation period, the sows were gradually fed with 1.0 and 2.0 kg/d lactation diet on day 1 and 2 of lactation, respectively; daily feed intake of lactating sows was 2.5 kg from day 3 to 6, and 3.0 kg from day 7 to 10. The sows were fed with lactation diet *ad libitum* from day 11 of lactation. Sows were fed twice daily at 7:30 and 14:30, and the amount of food consumed was recorded. Water was freely available throughout the experimental period. The temperature and humidity in the house were 22–24°C and 65–70%.

### 2.3 Sample collection

Fresh fecal samples from 5 sows in each group were collected at the end of lactation, stored immediately at −20°C for further processing. The anterior luminal venous blood from 5 sows in each group was collected in the morning before feeding at the end of lactation, and serum was separated by centrifugation at 3,000 × g for 5 min at 4°C, and stored at −80°C for the further use.

### 2.4 Reproductive performance determination

The backfat thickness of sows at P2 point was measured by ultrasonic backfat indicator (Renco lean-meter; Renco Corporation, Minneapolis, USA) on day 85 of gestation, parturition and weaning. The changes of backfat at each stage were calculated. The number of total born piglets (alive), litter size and weight at birth, and the average individual weight at birth were recorded. The survival rate of the piglets at birth was calculated. At the end of the lactation, the litter weight and number of weaned piglets were recorded, then the average daily gain and survival rate of piglets were calculated.

### 2.5 Nutrient digestibility

The diet and feces samples collected during the experiment were dried at 65°C, ground and sieved through a 1 mm screen in diameter. The nutrients in diets and feces were analyzed for crude protein (26), ether extract (26), calcium (27) and phosphorus (27). Neutral detergent fiber (NDF), acid detergent fiber (ADF), cellulose and hemicellulose were determined based on the previous method (28). The nutrient digestibility was determined using acid insoluble ash (AIA).

### 2.6 Serum biochemical index determination

Serum samples were thawed at 4°C and mixed well before analysis. Glucose (GLU), total cholesterol (TC), triglyceride (TG), high-density lipoprotein cholesterol (HDL-C), and low-density lipoprotein cholesterol (LDL-C) in sow serum were measured using an automated serum biochemical analyzer (Au5800, Beckman Coulter, Brea, USA).

### 2.7 Serum metabolomics

Metabolite extraction: 100 μL serum sample and 500 μL extract solution (acetonitrile: methanol = 1:1) were subjected to whirlpool shock for 30 s, followed by ultrasound treatment in ice-water bath for 10 min, and kept at-20°C for 60 min. After centrifugation at 14,000 × g for 15 min at 4°C, 500 μL supernatant was transferred to a centrifuge tube and dried in a vacuum concentrator to extract the metabolites, which was re-dissolved in 160 μL 50% acetonitrile. The solution was oscillated in a whirlpool for 30 s, ultrasonicated in ice-water bath for 10 min, centrifuged at 14,000 × g and 4°C for 15 min. Then 120 μL supernatant was put into a sample bottle for liquid chromatography/mass spectrum (LC/MS) analysis.

LC/MS analysis: The ultra-high-performance liquid chromatography (UHPLC) was performed using an ACQUITY UPLC I-Class PLUS instrument (Waters, Milford, USA), equipped with an ACQUITY UPLC HSS T3 Amide column. The mobile phase consisted of ultrapure water containing 0.1% formic acid (A) and acetonitrile containing 0.1% formic acid (B). The analysis was carried with elution gradient as follows: 0–0.25 min, 98% A and 2% B; 0.25–13.10 min, 2% A and 98% B; 13.10–15.00 min, 98% A and 2% B. The column temperature was 25°C. The auto-sampler temperature was 4°C, and the injection volume was 1 μL.

Mass spectral data were acquired using the MSe mode of the acquisition software (MassLynx V4.2) under the control of Xevo G2-XS QTOF high resolution mass spectrometer (Waters, Milford, USA). The MS parameters were as follows: scan mode (Orbitrap), ion spray voltage in ESI^+^ = 5,500 V, ion spray voltage in ESI^−^ = −4,500 V, nebulizer gas = 55 psi, heater gas = 65 psi, curtain gas = 35 psi, capillary temperature = 600°C, declustering potential = 60 V, ion spray voltage floating (ISVF) in positive or negative modes = 5,000 or-4,000 V.

Peak extraction, peak alignment and other data were obtained by processing the original data collected by MassLynx V4.2 with Progenesis QI software, and identification was carried out based on the online METLIN database of Progenesis QI software and the self-built database of BMK.

Bioinformatic analysis of sequencing data: Principal component analysis (PCA) and partial least squares discriminant analysis (PLS-DA) were performed in the present study to analyze the differences in serum metabolites, and the variable importance projection (VIP) value was employed to screen potential metabolic markers. Briefly, VIP > 1 and *p* < 0.05 were defined as differences based on the test of PLS-DA. Moreover, the Kyoto Encyclopedia of Genes and Genomes (KEGG) database was used to screen the key metabolic pathways.

### 2.8 Data calculations

The parameters were calculated based on the following formulae:

$$\mathrm{Birth}\;\mathrm{survive}\;\mathrm{rate}\;\left(\%\right)=\frac{\mathrm{Number}\;\mathrm{of}\;\mathrm{born}\;\mathrm{alive}\;\mathrm{piglets}}{\mathrm{Number}\;\mathrm{of}\;\mathrm{total}\;\mathrm{born}\;\mathrm{piglets}}\times 100$$

$$\mathrm{Birth}\;\mathrm{interval}\;\left(\min\right)=\frac{\mathrm{Duration}\;\mathrm{of}\;\mathrm{farrowing}\;\left(\min\right)}{\mathrm{Number}\;\mathrm{of}\;\mathrm{total}\;\mathrm{born}\;\mathrm{piglets}}$$

$$\begin{array}{c} \mathrm{Backfat}\;\mathrm{thickness}\;\mathrm{gain}\\ \mathrm{during}\;\mathrm{late}\;\mathrm{gestation}\;\left(\mathrm{mm}\right) \end{array}=BF2\;\left(\mathrm{mm}\right)-BF1\;\left(\mathrm{mm}\right)$$

$$\begin{array}{c} \mathrm{Backfat}\;\mathrm{thickness}\;\mathrm{loss}\\ \mathrm{during}\;\mathrm{lactation}\;\left(\mathrm{mm}\right) \end{array}\;=BF3\;\left(\mathrm{mm}\right)-BF2\;\left(\mathrm{mm}\right)$$

$$\mathrm{Digestibility}\;\mathrm{of}\;\mathrm{nutrients}\;\left(\%\right)=100\times \left(1-\frac{A}{B}\times \frac{C}{D}\right)$$

where *BF1* is the sow backfat thickness on day 85 of gestation, *BF2* is the sow backfat thickness at farrowing, *BF3* is the sow backfat thickness at weaning, *A* is the content of AIA in the diet (%), *B* is the content of AIA in the feces (%), *C* is nutrient content in the feces (%), and *D* is the nutrient content in the diet (%) (29).

### 2.9 Data analysis

The statistical analyzes were performed using SPSS 26.0 Software (IBM, NYC, USA). One-way ANOVA was used to analyze the data, and the differences between means assessed by using Duncan’s test. Statistical results were presented as mean ± SEM (*n* = 8), and *p* < 0.05 was considered as statistical significance.

## 3 Results

### 3.1 Sow reproductive performance affected by CSSF

Table 3 indicated that 6% LFHSCSSF addition significantly increased number of total born (alive) piglets, litter weight at birth (*p* < 0.05), whereas three kinds of CSSF significantly decreased backfat thickness of sows during late gestation (*p* < 0.001), compared with the control group. Furthermore, CSSF additions had no significant effect on dry matter intake during late gestation (*p* = 0.304).

**Table 3:** Effects of different kinds of CSSF on sow productive performance during late gestation.

Item	A^1^	B^2^	C^3^	D^4^	SEM	*p*-value
Average daily dry matter intake, kg/d	2.98	2.91	2.98	3.00	0.018	0.304
Number of total born piglets	12.88 ^b^	15.00 ^ab^	14.75 ^ab^	17.25 ^a^	0.558	0.043
Number of born alive piglets	11.88 ^b^	14.25 ^ab^	13.25 ^ab^	15.50 ^a^	0.482	0.045
Birth survive rate, %	91.92	95.56	90.68	90.04	1.222	0.399
Litter weight at birth, kg	14.59 ^b^	16.24 ^ab^	17.14 ^ab^	18.86 ^a^	0.553	0.041
Body weight at birth, kg	1.26 ^a^	1.14 ^b^	1.30 ^a^	1.22 ^ab^	0.020	0.023
Birth interval, min	11.96	11.65	9.15	9.80	0.770	0.513
Sow backfat thickness, mm						
On d 85 of gestation	16.88	17.00	17.00	17.75	0.234	0.553
At farrowing	22.00 ^a^	20.38 ^ab^	18.75 ^b^	19.13 ^b^	0.353	0.002
Backfat thickness gain during late gestation	5.13 ^a^	3.38 ^b^	1.75 ^c^	1.38 ^c^	0.306	< 0.001

^1^A: the basal diet. ^2^B: 6% HFLSCSSF added in the basal diet to replace corn meal and wheat bran. ^3^C: 6% MFMSCSSF added in the basal diet to replace corn meal and wheat bran. ^4^D: 6% LFHSCSSF added in the basal diet to replace corn meal and wheat bran. ^a–c^The different lowercase letters in the same row indicate significant differences (*p* < 0.05), whereas the same lowercase letters or without lowercase letters in the same row indicate insignificant differences (*p* > 0.05).

As shown in Table 4, litter size of weaned piglets in group C was higher than that in groups A and D (*p* < 0.05), body weight and average daily gain of weaned piglets in group D were higher than that in groups A and C (*p* < 0.05), sow backfat thickness at weaning in group A was thicker than that in groups B and C (*p* < 0.01), sow backfat loss during lactation from high to low was: group B > group A > groups C and D (*p* < 0.001). In addition, CSSF additions had insignificant effect on estrous interval (*p* = 0.344) and dry matter intake during lactation (*p* = 0.212).

**Table 4:** Effects of different kinds of CSSF on sow productive performance during lactation.

Item	A^1^	B^2^	C^3^	D^4^	SEM	*p*-value
Average daily dry matter intake, kg/d	5.42	5.31	5.39	5.62	0.054	0.212
Litter size of weaned piglets	11.00 ^b^	12.00 ^ab^	12.38 ^a^	11.13 ^b^	0.209	0.044
Body weight of weaned piglets, kg	6.07 ^c^	6.61 ^ab^	6.14 ^bc^	6.69 ^a^	0.098	0.038
Average daily gain of piglet, kg/d	0.19 ^b^	0.22 ^a^	0.19 ^b^	0.22 ^a^	0.004	0.009
Estrous interval, day	5.00	5.25	5.25	5.63	0.121	0.344
Sow backfat thickness at weaning, mm	17.25 ^a^	14.13 ^b^	14.75 ^b^	15.88 ^ab^	0.356	0.005
Sow backfat loss during lactation, mm	4.75 ^b^	6.25 ^a^	4.00 ^c^	3.25 ^c^	0.258	< 0.001

^1^A: the basal diet. ^2^B: 6% HFLSCSSF added in the basal diet to replace corn meal and wheat bran. ^3^C: 6% MFMSCSSF added in the basal diet to replace corn meal and wheat bran. ^4^D: 6% LFHSCSSF added in the basal diet to replace corn meal and wheat bran. ^a–c^The different lowercase letters in the same row indicate significant differences (*p* < 0.05), whereas the same lowercase letters or without lowercase letters in the same row indicate insignificant differences (*p* > 0.05).

### 3.2 Nutrient digestibility of lactating sows affected by CSSF

Table 5 showed that the addition of three kinds of CSSF to the diets of lactating sows in groups B, C and D significantly increased nutrient digestibility, compared with group A (*p* <0.001). The digestibility of DM was highest in group D, followed by group B, group C and group A (*p* < 0.001). The digestibility of CP was highest in group B, followed by group D, group C and group A (*p* < 0.001). The digestibility of EE and energy was highest in group D, followed by group B, group C and group A (*p* < 0.001). The digestibility of Ca was highest in groups C and D, followed by group B and group A (*p* < 0.001). The digestibility of P was highest in groups B, C and D, followed by group A (*p* < 0.001). The digestibility of NDF was highest in group C, followed by group B, group D and group A (*p* < 0.001). The digestibility of cellulose was highest in group D, followed by group B, group C and group A (*p* < 0.001). The digestibility of hemicellulose was highest in group C, followed by group B, group D and group A (*p* < 0.001). Based on the above results, three kinds of CSSF additions in the diets of lactating sows significantly increased nutrient digestibility (*p* < 0.001).

**Table 5:** Effects of different CSSF on nutrient digestibility of lactating sows (%).

Item	A^1^	B^2^	C^3^	D^4^	SEM	*p*-value
DM	72.17 ^c^	80.91 ^b^	80.48 ^b^	83.67 ^a^	1.297	< 0.001
CP	73.22 ^d^	82.83 ^a^	79.74 ^c^	80.50 ^b^	1.077	< 0.001
EE	64.28 ^c^	81.26 ^b^	81.99 ^b^	90.39 ^a^	2.929	< 0.001
Energy	71.42 ^c^	81.01 ^b^	80.92 ^b^	83.62 ^a^	1.402	< 0.001
Ca	34.41 ^c^	44.20 ^b^	46.69 ^a^	45.47 ^ab^	1.488	< 0.001
P	46.10 ^b^	62.34 ^a^	62.05 ^a^	63.68 ^a^	2.200	< 0.001
NDF	70.27 ^c^	79.92 ^b^	83.48 ^a^	80.24 ^b^	1.525	< 0.001
ADF	29.95 ^d^	63.40 ^a^	54.38 ^c^	56.83 ^b^	3.828	< 0.001
Cellulose	34.77 ^d^	65.10 ^b^	61.85 ^c^	66.99 ^a^	3.944	< 0.001
Hemicellulose	76.15 ^c^	84.35 ^b^	88.14 ^a^	84.07 ^b^	1.328	< 0.001

DM, dry matter; CP, crude protein; EE, ether extract; Ca, calcium; P, phosphorus; NDF, neutral detergent fiber; ADF, acid detergent fiber. ^1^A: the basal diet. ^2^B: 6% HFLSCSSF added in the basal diet to replace corn meal and wheat bran. ^3^C: 6% MFMSCSSF added in the basal diet to replace corn meal and wheat bran. ^4^D: 6% LFHSCSSF added in the basal diet to replace corn meal and wheat bran. ^a–d^The different lowercase letters in the same row indicate significant differences (*p* < 0.05), whereas the same lowercase letters or without lowercase letters in the same row indicate insignificant differences (*p* > 0.05).

### 3.3 Serum biochemical parameters of lactating sows affected by CSSF

**Figure 1:**
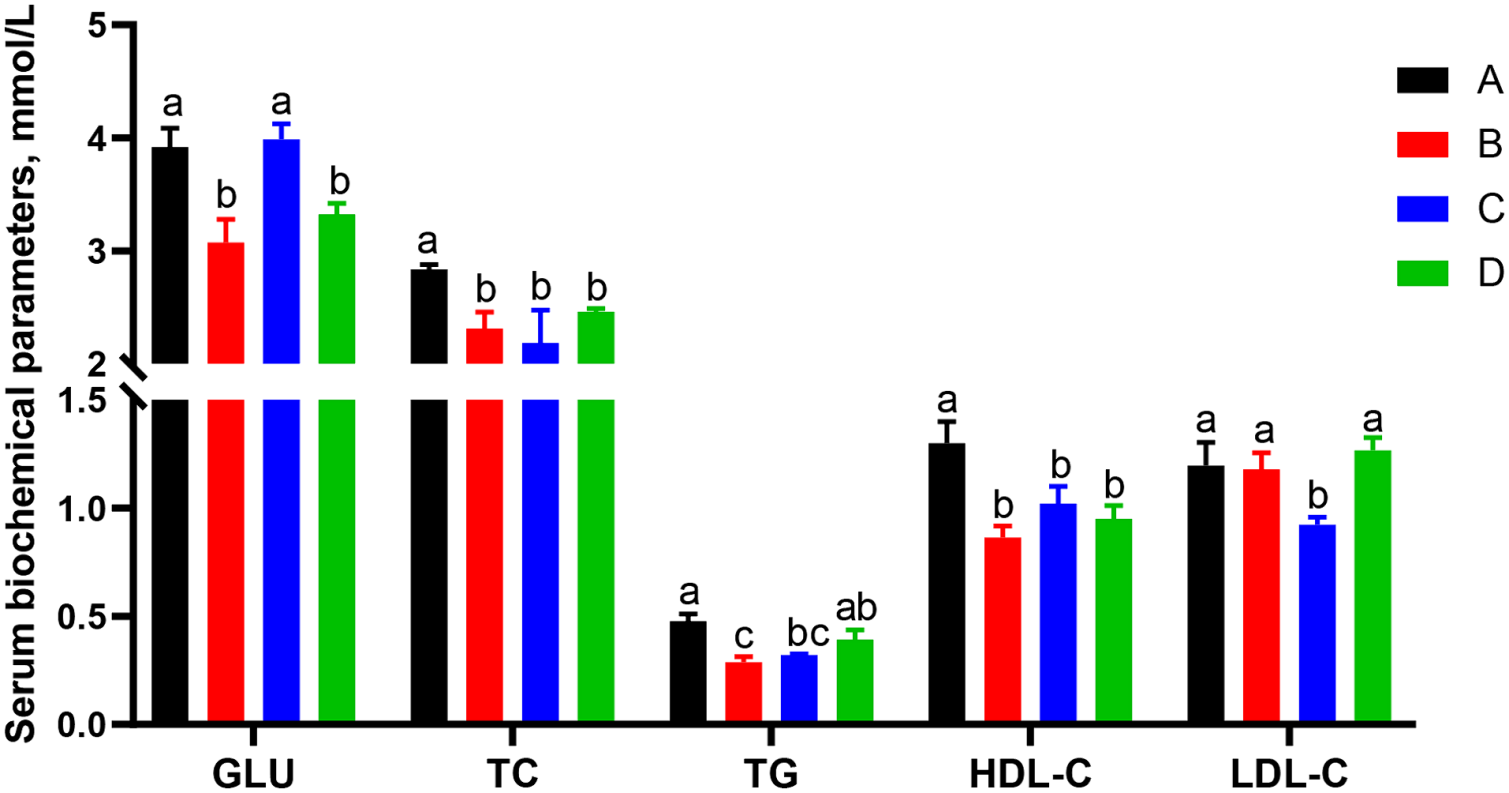
Effects of different kinds of CSSF on serum biochemical parameters of sows on d 25 of lactation. GLU, glucose; TC, total cholesterol; TG, total triglycerides; HDL-C, high-density lipoprotein cholesterol; LDL-C, low-density lipoprotein cholesterol. A, the basal diet; B–D, 6% HFLSCSSF, 6% MFMSCSSF and 6% LFHSCSSF added in the basal diet to replace some parts of corn meal and wheat bran, respectively. ^a–c^The different lowercase letters on each bar indicate significant difference (*p* < 0.05), whereas the same lowercase letters on each bar indicate insignificant difference (*p* > 0.05).

Effects of different CSSF on serum biochemical parameters of sows on day 25 of lactation were shown in Figure 1. The serum TC and HDL-C contents in groups B, C and D were significantly lower than that in group A (*p* < 0.05); serum GLU contents in groups B and D were significantly lower than that in groups A and C (*p* < 0.05); serum TG contents in groups A was significantly higher than that in groups B and C (*p* < 0.05), but without significant difference between group A and D, group C and D or group B and C (*p* > 0.05); serum LDL-C content in group C was significantly lower than that in other groups (*p* < 0.05). It was concluded that three kinds of CSSF additions in the diets of lactating sows were able to decrease the serum contents of most biochemical parameters such as GLU, TC, TG, and HDL-C.

### 3.4 Effect of CSSF on serum metabolomics for lactating sows

**Figure 2:**
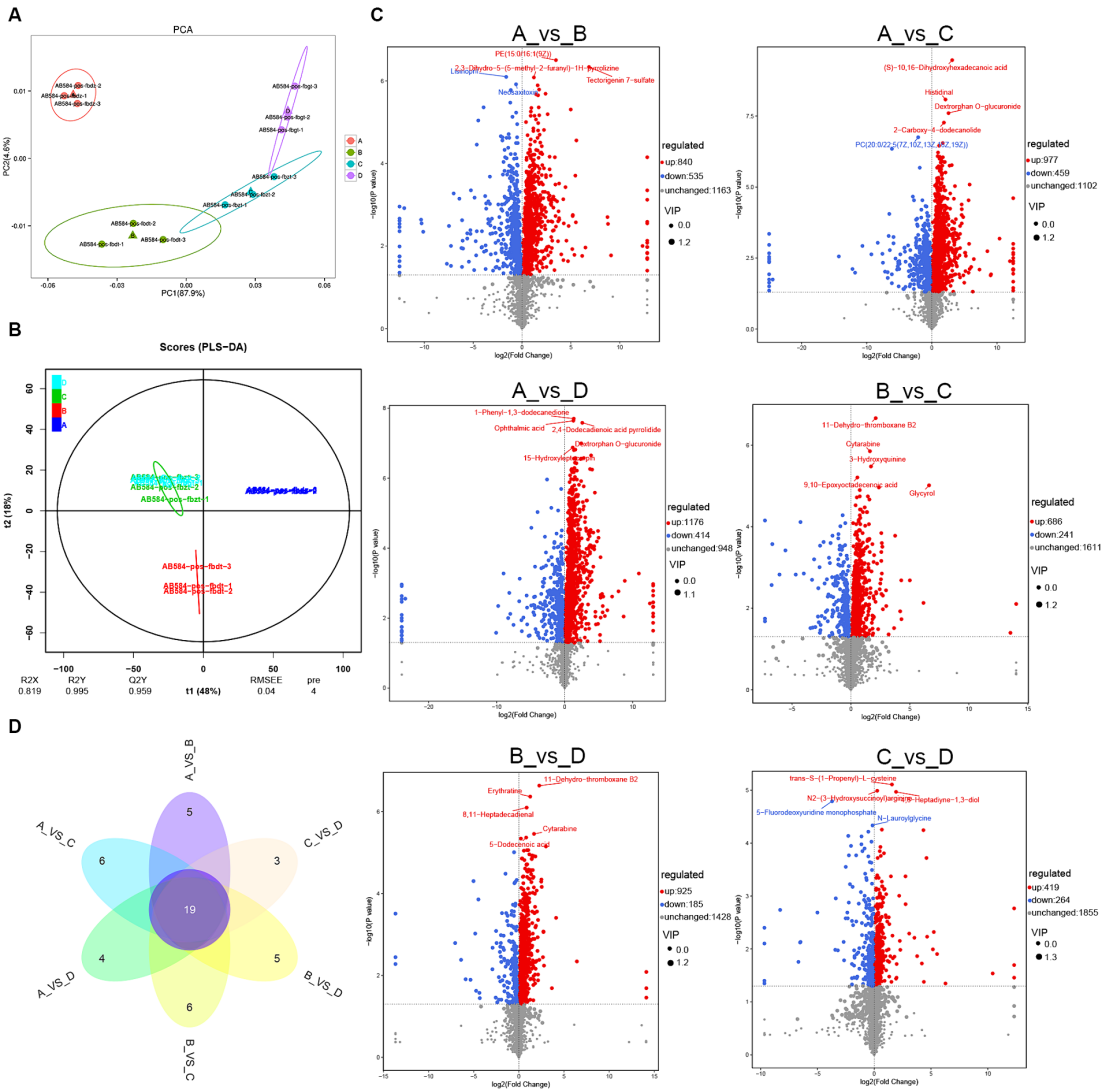
Serum metabolites analysis of lactating sows: **(A)** PCA analysis of metabolites; **(B)** PLS-DA model analysis for each group; **(C)** volcanic map of differential metabolites in both-group comparisons such as group A vs. B, group A vs. C, group A vs. D, group B vs. C, group B vs. D and group C vs. D, respectively; **(D)** Veen map of differential metabolites in both-group comparisons. A, the basal diet; B–D, 6% HFLSCSSF, 6% MFMSCSSF and 6% LFHSCSSF added in the basal diet to replace some parts of corn meal and wheat bran, respectively. The serial numbers of 12 serum samples are as follows: group A: AB584-pos-fbdz-1 (A1), AB584-pos-fbdz-2 (A2), AB584-pos-fbdz-3 (A3); group B: AB584-pos-fbdt-1 (B1), AB584-pos-fbdt-2 (B2), AB584-pos-fbdt-3 (B3); group C: AB584-pos-fbzt-1 (C1), AB584-pos-fbzt-2 (C2), AB584-pos-fbzt-3 (C3); group D: AB584-pos-fbgt-1 (D1), AB584-pos-fbgt-2 (D2), AB584-pos-fbgt-3 (D3).

In order to investigate the effects of different CSSF on the metabolism of lactating sows, 3 serum samples in each group were analyzed by non-target metabolomics using UHPLC-QTOF-MS. The PCA score plot demonstrated good stability and repeatability of the serum samples in each group, in which PC1 and PC2 axes explained 87.9 and 4.6% of the variation, respectively (Figure 2A). In addition, the PLS-DA model (Figure 2B) showed that the model parameters of the serum (R^2^X = 0.819, R^2^Y = 0.995, Q^2^Y = 0.959) were all greater than 0.8, indicating a high accuracy of the PLS-DA model and strong predictive ability. Furthermore, there were significant differences among four groups, indicating that three kinds of CSSF significantly affected the serum metabolism of lactating sows.

A total of 2,538 metabolites were detected in the serum of 12 lactating sows. The metabolites were screened for comparisons between both groups such as group A vs. B, group A vs. C, group A vs. D, group B vs. C, group B vs. D, group C vs. D, resulting in the identification of 1,375, 1,436, 1,590, 927, 1,110 and 683 differential metabolites, respectively. Compared with group A, 840 metabolites were up-regulated and 535 metabolites were down-regulated in group B, 977 metabolites were up-regulated and 459 metabolites were down-regulated in group C, 1,176 metabolites were up-regulated and 414 metabolites were down-regulated in group D. Compared with group B, 686 metabolites were up-regulated and 241 metabolites were down-regulated in group C, 925 metabolites were up-regulated and 185 metabolites were down-regulated in group D. Compared with group C, there were 419 up-regulated metabolites and 264 down-regulated metabolites in group D. Furthermore, volcanic maps were used to analyze the differential metabolites after the different treatments, and the top 5 metabolites with the most significant differences in each treatment group were labeled accordingly (Figure 2C).

Veen diagram analysis of the differential metabolites in both-group comparisons (group A vs. B, group A vs. C, group A vs. D, group B vs. C, group B vs. D, and group C vs. D) indicated that there were a total of 19 common differential metabolites (Figure 2D). The specific metabolites were shown in Table 6. Compared to group A, the addition of different CSSF in the diet of lactating sows significantly increased the levels of metabolites such as 4-pyridoxate, L-carnitine, cis-2-methylaconitate, phosphatidyl cholines (PC20, (20:2(11Z,14Z)/22:6(4Z,7Z,10Z,13Z,16Z,19Z)); PC18, (18:1(11Z)/22:5(4Z,7Z,10Z,13Z,16Z))), 4-pyridoxic acid, 11-dehydro-thromboxane B2, traumatic acid, androsterone glucuronide and L-tyrosine (*p* <0.05), whereas decreased the levels of cyclic GMP-AMP and 2-n-propyl-4-oxopentanoic acid (*p* <0.05). Additionally, lumichrome and 3-methyldioxyindole levels were significantly decreased in group B, and increased in groups C and D, compared with group A (*p* <0.05).

**Table 6:** The common differential metabolites in both-group comparisons.

Metabolites	Major metabolic pathways		Group B^2^ vs. Group A^1^	Group C^3^ vs. Group A^1^	Group D^4^ vs. Group A^1^
	Pathway ID	Pathway			
4-Pyridoxate	map00750	Vitamin B6 metabolism	Up	Up	Up
L-Carnitine	map04976	Bile secretion	Up	Up	Up
cis-2-Methylaconitate	map00640	Propanoate metabolism	Up	Up	Up
PC20	map00592	Alpha-linolenic acid metabolism	Up	Up	Up
4-Pyridoxic acid	map00750	Vitamin B6 metabolism	Up	Up	Up
11-Dehydro-thromboxane B2	map00590	Arachidonic acid metabolism	Up	Up	Up
Traumatic acid	map00592	Alpha-linolenic acid metabolism	Up	Up	Up
PC18	map00592	Alpha-linolenic acid metabolism	Up	Up	Up
Androsterone glucuronide	map00140	Steroid hormone biosynthesis	Up	Up	Up
L-Tyrosine	map00400	Phenylalanine, tyrosine and tryptophan biosynthesis	Up	Up	Up
Biliverdin	map00860	Porphyrin and chlorophyll metabolism	Up	Up	Up
Heteroxanthin	map00232	Caffeine metabolism	Up	Up	Up
2-Polyprenyl-3-methyl-5-hydroxy-6-methoxy-1,4-benzoquinone	map00130	Ubiquinone and other terpenoid-quinone biosynthesis	Up	Up	Up
Coniferol	map00940	Phenylpropanoid biosynthesis	Up	Up	Up
L-erythro-tetrahydrobiopterin	map00790	Folate biosynthesis	Up	Up	Up
cyclic GMP-AMP	map05131	Shigellosis	Down	Down	Down
2-n-Propyl-4-oxopentanoic acid	map00982	Drug metabolism-cytochrome P450	Down	Down	Down
Lumichrome	map00740	Riboflavin metabolism	Down	Up	Up
3-Methyldioxyindole	map00380	Tryptophan metabolism	Down	Up	Up

PC20 = phosphatidyl cholines (20:2(11Z,14Z)/22:6(4Z,7Z,10Z,13Z,16Z,19Z)); PC18 = phosphatidyl cholines (18:1(11Z)/22:5(4Z,7Z,10Z,13Z,16Z)). ^1^Group A: the basal diet. ^2^Group B: 6% HFLSCSSF added in the basal diet to replace corn meal and wheat bran. ^3^Group C: 6% MFMSCSSF added in the basal diet to replace corn meal and wheat bran. ^4^Group D: 6% LFHSCSSF added in the basal diet to replace corn meal and wheat bran.

### 3.5 KEGG pathway enrichment analysis of differential metabolites

**Figure 3:**
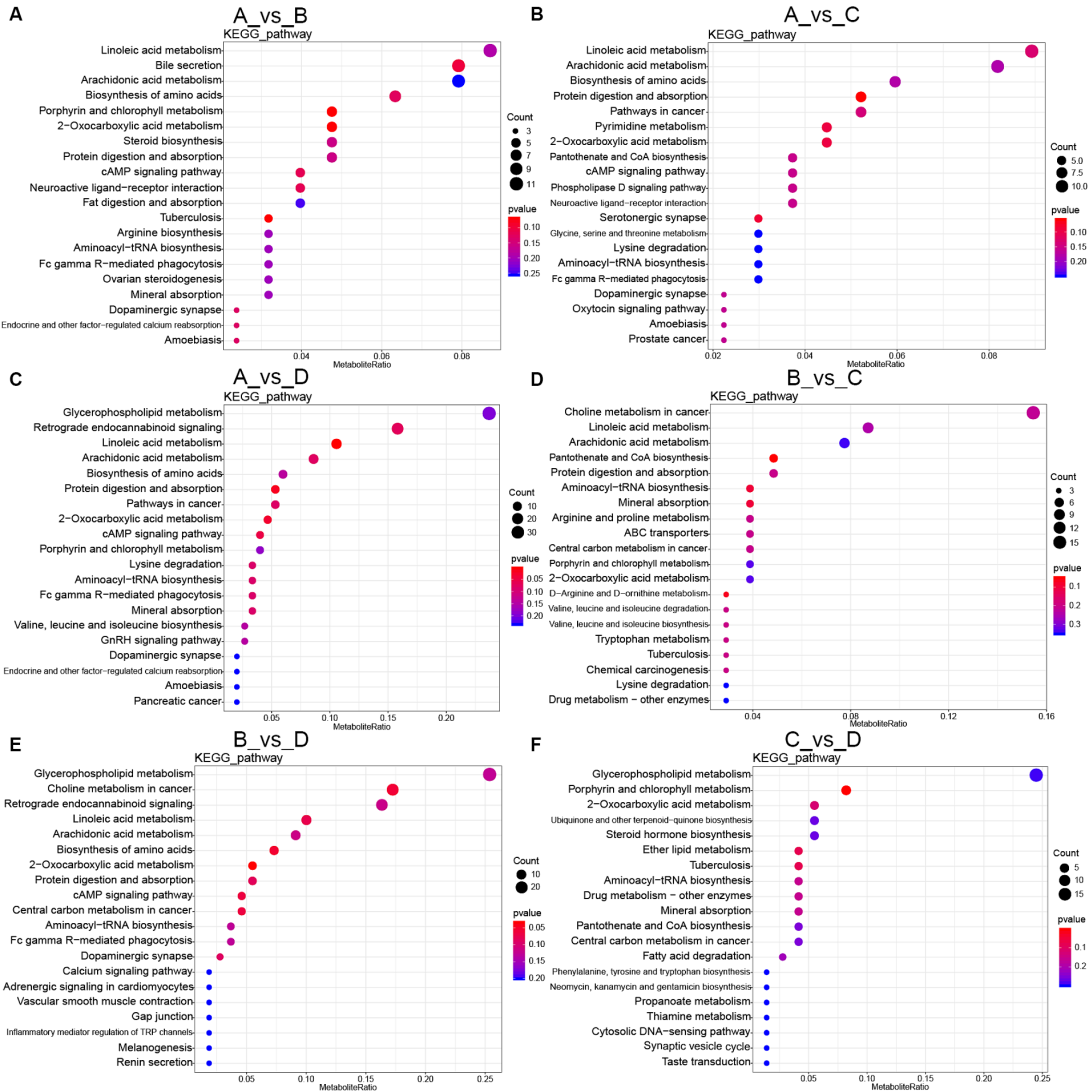
KEGG pathway enrichment of differential metabolites: **(A–F)** Different metabolites were enriched in KEGG pathway in both-group comparisons (group A vs. B, group A vs. C, group A vs. D, group B vs. C, group B vs. D, group C vs. D). A, the basal diet; B–D, 6% HFLSCSSF, 6% MFMSCSSF and 6% LFHSCSSF added in the basal diet to replace some parts of corn meal and wheat bran, respectively.

The analysis for the enrichment of metabolic pathways was shown in Figure 3. Compared with group A, the main common metabolic pathways included linoleic acid metabolism, arachidonic acid metabolism, biosynthesis of amino acids, protein digestion and absorption in both-group comparisons (group A vs. B, group A vs. C, group A vs. D). In addition, compared with group A, the unique metabolic pathways were bile secretion in group B; glycine, serine and threonine metabolisms in group C; glycerophospholipid metabolism in group D.

Compared with group B, the primarily affected metabolic pathways were choline metabolism in cancer, pantothenate and CoA biosynthesis in group C; glycerophospholipid metabolism, choline metabolism in cancer in group D. Furthermore, the main metabolic pathways affected in group D included glycerophospholipid metabolism, porphyrin and chlorophyll metabolism, 2-oxocarboxylic acid metabolism, ubiquinone and other terpenoid-quinone biosynthesis and steroid hormone biosynthesis in comparison with group C.

Additionally, the KEGG analysis of the common differential metabolites in both-group comparisons (group A vs. B, group A vs. C, group A vs. D, group B vs. C, group B vs. D, group C vs. D) revealed that there were 19 common differential metabolites primarily concentrated in the metabolisms including alpha-linolenic acid, arachidonic acid, tryptophan and propionate; steroid hormone synthesis; bile secretion; phenylalanine, tyrosine and tryptophan biosynthesis. Therefore, the different kinds of CSSF regulated lipid, amino acid, cofactors and vitamins metabolisms to improve reproductive performance of lactating sows.

### 3.6 Correlation analysis between serum differential metabolites and potential influential factors

**Figure 4:**
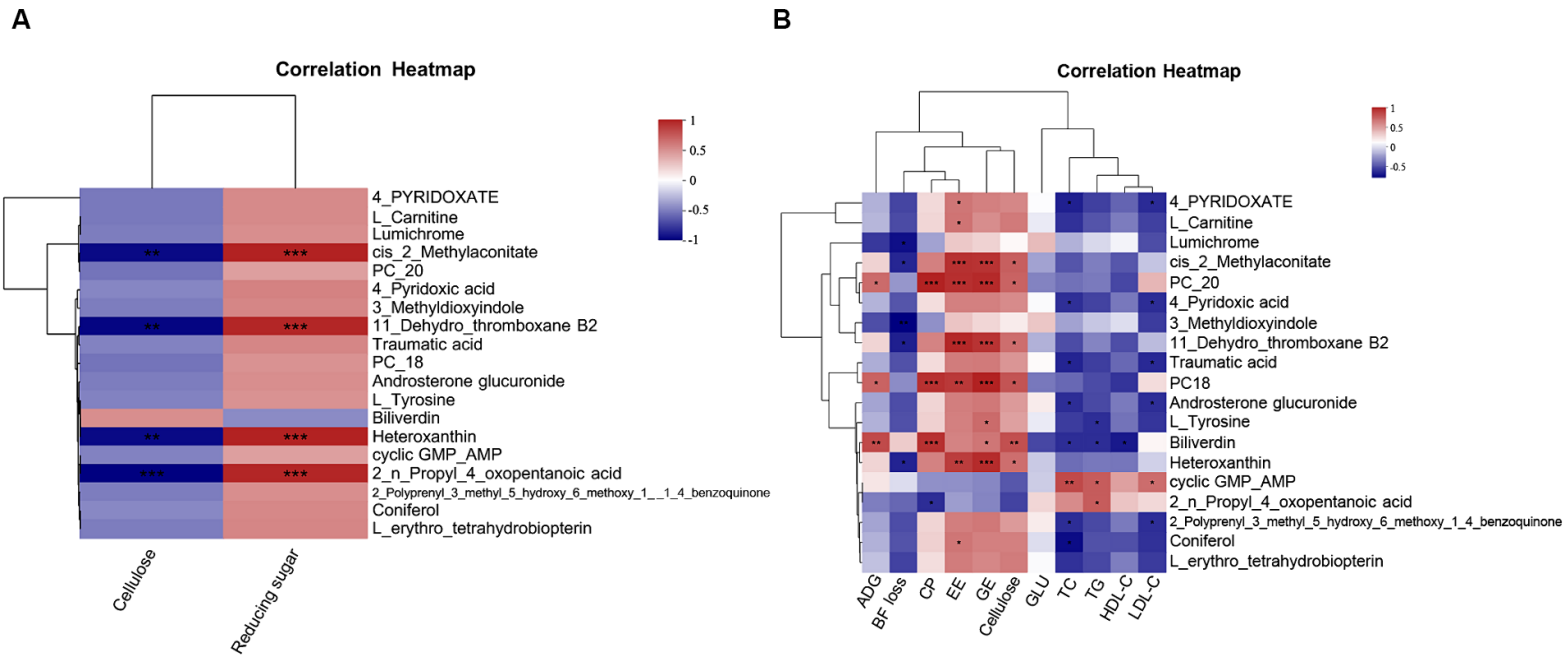
Correlation analysis: **(A)** Correlation analysis between serum differential metabolites and the fiber or reducing sugar content in three kinds of CSSF. **(B)** Correlation analysis between serum differential metabolites and ADG, BF loss, nutrient digestibility or serum biochemical parameters. Cellulose, cellulose content in three kinds CSSF; Reducing sugar, reducing sugar content in three kinds CSSF; PC20: phosphatidyl cholines (20:2(11Z,14Z)/22:6(4Z,7Z,10Z,13Z,16Z,19Z)); PC18: phosphatidyl cholines (18:1(11Z)/22:5(4Z,7Z,10Z,13Z,16Z)); ADG, average daily gain of piglet; BF loss, backfat thickness loss of sows during lactation; CP, EE and GE represent crude protein, ether extract, energy digestibility of lactating sows, respectively; GLU, glucose; TC, total cholesterol; TG, total triglycerides; HDL-C, high-density lipoprotein cholesterol; LDL-C, low-density lipoprotein cholesterol. *Indicates *p* < 0.05, ** indicates *p* < 0.01, *** indicates *p* < 0.001.

To investigate the effect of CSSF on serum metabolites, we conducted a correlation analysis between the fiber or reducing sugar content in CSSF and serum differential metabolites. It was found that serum cis-2-methylaconitate, 11-dehydro-thromboxane B2, heteroxanthin, and 2-n-propyl-4-oxopentanoic acid levels were significantly positively correlated with the reducing sugar content in CSSF (*p* < 0.05), whereas they were significantly negatively correlated with the fiber content in CSSF (*p* < 0.05) (Figure 4A).

Spearman correlation analysis was used to analyze the associations between the common differential metabolites in serum and average daily gain (ADG) of piglets, backfat thickness loss (BF loss), nutrient digestibility and serum biochemical parameters of sows, respectively (Figure 4B). ADG showed a significant positive correlation with the levels of metabolites such as PC20, PC18 and biliverdin (*p* < 0.05). Lumichrome, cis-2-methylaconitate, 3-methyldioxyindole, 11-dehydro-thromboxane B2 and heteroxanthin levels were significantly negatively correlated with BF loss (*p* < 0.05). The CP digestibility was positively correlated with the levels of metabolites such as PC20, PC18 and biliverdin (*p* < 0.001), but negatively correlated with 2-n-propyl-4-oxopentanoic acid level (*p* < 0.05). The digestibility of EE showed positive correlation with the levels of metabolites such as 4-pyridoxate, L-carnitine, cis-2-methylaconitate, PC20, PC18, 11-dehydro-thromboxane B2, heteroxanthin and coniferol (*p* < 0.05). Cis-2-methylaconitate, PC20, PC18, 11-dehydro-thromboxane B2, biliverdin and heteroxanthin levels showed positive correlation with the digestibility of energy and cellulose (*p* < 0.05). Additionally, serum TC and LDL-C contents had significant positive correlation with the level of cyclic GMP-AMP metabolite in serum (*p* < 0.05), but negative correlation with the levels of metabolites such as 4-pyridoxate, 4-pyridoxic acid, traumatic acid, androsterone glucuronide and 2-polyprenyl-3-methyl-5-hydroxy-6-methoxy-1-4-benzoquinone (*p* < 0.05). Serum TG content was significantly positively correlated with cyclic GMP-AMP and 2-n-propyl-4-oxopentanoic acid levels (*p* < 0.05), but negatively correlated with L-tyrosine and biliverdin levels (*p* < 0.05). Serum HDL-C content showed significant negative correlation with biliverdin level (*p* < 0.05). It was concluded that the different kinds of CSSF play important roles through regulating metabolisms and biosynthesis of metabolites.

## 4 Discussion

Dietary fiber can satisfy the full feeling of sows to prevent excessive energy intake during gestation and regulate reproductive performance (30, 31). Previous studies showed that feeding sow diets rich in wheat straw (32), alfalfa (33) and *radix puerariae* residue (34) during gestation could improve the reproductive performance of sows. In addition, supplementation of sow diets with low, medium and high fiber levels increased the total number of born (alive) piglets (35). This study demonstrated that feeding sows different CSSF diets during late gestation and lactation increased the total number of born (alive) piglets and the average daily gain of piglets, in which LFHSCSSF indicated the best effects, maybe due to its low-fiber and high-saccharification result to provide more available energy resource. Backfat thickness is an indicator of the body condition and energy reserve of sows (36). Excessive or insufficient backfat thickness can affect sow production and estrus cycle (37); therefore, maintaining backfat thickness within the appropriate range is important for reproductive performance of sows. Feeding a high-fiber diet during gestation reduced backfat gain of sows (38), which is consistent with this study. The backfat loss during lactation may be related to the low backfat gain during gestation (39). This study indicated that MFMSCSSF and LFHSCSSF additions could prevent backfat thickness loss for lactating sows, inferring that both of them had the ability to regulate lipid metabolism beneficial for sow reproductive performance.

High-fiber diets have a negative impact on nutrient digestibility of sows (39). The previous study showed that dietary fiber reduced the contact time between chyme and intestinal digestive enzymes, thus reducing the digestion and absorption of nutrients (40). However, recent researches reported that fiber diets could improve the digestibility of crude protein, ether extract and neutral detergent fiber for sows (20, 41). Additionally, fiber-rich products were found to improve the apparent nutrient digestibility for sows and have the potential to replace expensive grains (42). In this study, the nutrient digestibility was improved by sows in different CSSF groups, due to the different physicochemical properties of CSSF that result in changes in the digestion and absorption of nutrients (43).

Dietary fiber is known to reduce fat accumulation and ameliorate obesity (44). The previous studies indicated that bamboo powder could reduce total cholesterol and triglyceride levels in the serum of sows (45), and sugar beet pulp and wheat bran could decrease serum cholesterol levels (46). This study showed that the addition of CSSF to sow diets reduced the levels of serum triglycerides, cholesterol and high-density lipoprotein cholesterol. This may be due to dietary fiber in CSSF binding cholesterol and bile acids to enhance their excretions or inhibit absorptions (47). These results suggested that CSSF had the potential to regulate serum lipid metabolism.

Through metabonomics analysis of lactating sows, it was discovered that feeding different kinds of CSSF significantly increased the expressions of 4-pyridoxic acid, PC20, PC18 and L-tyrosine, while significantly decreased the expressions of 2-n-propyl-4-oxopentanoic acid and cyclic GMP-AMP. PC is an important component of cell membranes, which can improve lipid metabolism and promote recovery of liver function (48). When PC is deficient in the body, it can lead to fat accumulation and lipid metabolism disorders (49). The up-regulation of PC expression by CSSF supplementation in this study suggested that CSSF could protect body health by regulating lipid metabolism. It was found that feeding PC and high-fat diets to mice significantly reduced the total cholesterol content in the liver compared to feeding high-fat diets alone (50), which was verified in this study. 4-Pyridoxic acid is the final product of vitamin B6 catabolism, and the lack of vitamin B6 in the body can result in inflammation, anemia, depression and insanity (51). The previous study demonstrated that consumption of fiber-rich chickpeas and hummus could increase the levels of 4-pyridoxine and total vitamin B6 in human serum (52), in agreement with study. L-tyrosine is converted from phenylalanine through the action of phenylalanine hydroxylase, which can use dopamine and dopamine to generate norepinephrine to promote lipolysis and fat mobilization (53, 54). It was showed that the addition of inulin to the diet increased level of L-tyrosine in the blood (55). In this study, the expression of L-tyrosine in the serum of sows fed with different CSSF was increased, and the thickness of backfat was decreased, indicating that CSSF could regulate body health by promoting fat degradation. Moreover, a fiber-rich diet could enhance lipid metabolism (56), amino acid metabolism (57), and cofactor and vitamin metabolism (58). The KEGG enrichment analysis performed in this study emphasized these metabolic pathways, suggesting that CSSF could maintain body homeostasis by regulating lipid metabolism, amino acid metabolism, cofactor and vitamin metabolism.

Cis-2-Methylaconitate is a primary metabolite directly involved in the growth, development and reproduction of organisms (59). 11-Dehydro-thromboxane B2 is a thromboxane metabolite, and elevated levels are associated with hyperglycemia, hypercholesterolemia, and cardiovascular disease (60). We found that the levels of cis-2-methylaconitate and 11-dehydro-thromboxane B2 were significantly positively correlated with the content of reducing sugar in CSSF, while they were significantly negatively correlated with the content of cellulose in CSSF. This demonstrates that CSSF could provide optimal levels of energy and dietary fiber for animal production as well as regulate glucose and cholesterol metabolism to improve animal health. Furthermore, this study showed that the levels of PC20, PC18 and biliverdin were significantly positively correlated with ADG and nutrient digestibility for lactating sows, while negatively correlated with serum biochemical indicators; however, cyclic GMP-AMP and 2-n-propyl-4-oxopentanoic acid levels showed the opposite results. PC has been found to inhibit the activities of lecithin cholesterol acyltransferase, acylcholesterol acyltransferase and lipoprotein lipase, thereby reducing the levels of total cholesterol and triglycerides in the body (61). Biliverdin is a product of heme catabolism with antioxidant, anti-inflammatory and anti-apoptotic effects (62). Furthermore, cyclic GMP-AMP is a second messenger that recognizes intracellular DNA and viral infections (63). Inhibition of cyclic GMP-AMP can effectively reduce inflammatory responses and alleviate oxidative stress-induced damage (64). Overall, these findings suggested that different kinds of CSSF involved in lipid metabolism, amino acid metabolism, co-factor and vitamin metabolism, resulting in low levels of total cholesterol and triglycerides in serum as well as low backfat thickness loss for lactating sows, thereby promoting sow health.

## 5 Conclusion

Three kinds of CSSF were prepared to partially replace corn meal and wheat bran in the diets of sows during late gestation and lactation. It was indicated that CSSF additions increased nutrient digestibility and reproductive performances of sows. This study provided a strategy for exploring straw resources to solve feedstuffs shortage and improve reproductive performance of sows by regulating lipid metabolisms.

## Data availability statement

The original contributions presented in the study are included in the article, further inquiries can be directed to the corresponding author.

## Ethics statement

All protocols in this experiment were approved by the Animal Care and Use Ethics Committee of Henan Agricultural University (SKLAB-B-2010-003-01), and conducted in compliance with the relevant regulations and guidelines. The study was conducted in accordance with the local legislation and institutional requirements.

## Author contributions

ML: Data curation, Formal analysis, Methodology, Writing – original draft. CL: Investigation, Resources, Software, Writing – original draft. JS: Data curation, Writing – original draft. PW: Investigation, Project administration, Supervision, Writing – review & editing. JC: Conceptualization, Investigation, Writing – review & editing. XX: Investigation, Methodology, Writing – review & editing. LW: Methodology, Resources, Writing – original draft. SJ: Formal analysis, Validation, Writing – original draft. XL: Data curation, Formal analysis, Writing – review & editing. QY: Funding acquisition, Project administration, Supervision, Writing – review & editing. QZ: Data curation, Validation, Writing – original draft. XD: Data curation, Formal analysis, Validation, Writing – original draft. FL: Resources, Validation, Writing – original draft.
